# Latent class analysis of depressive symptoms and associations with suicidal thoughts, plans, and attempts among a large national sample

**DOI:** 10.1017/S0033291724002009

**Published:** 2024-09

**Authors:** Annabelle M. Mournet, Evan M. Kleiman

**Affiliations:** Department of Psychology, Rutgers, The State University of New Jersey, Piscataway, NJ, USA

**Keywords:** adult, depression, latent class analysis, NSDUH, suicide

## Abstract

**Background:**

Depression is strongly associated with risk for suicidal behaviors. However, depression is a highly heterogeneous condition (i.e. there are more than 200 combinations of DSM-5-TR depressive symptoms to correspond to a depression diagnosis). Limited research to date has taken an empirical approach to see how people cluster together based on their classification of depressive symptoms and whether people in certain classes are more likely to report suicide outcomes than other classes. This analysis leverages the National Survey on Drug Use and Health and examines classes of depressive symptoms to explore differences in suicide-related outcomes by class among adults endorsing depressive symptoms (*n* = 41 969).

**Methods:**

We used latent class analysis (LCA) to identify classes of individuals’ DSM-5 depressive symptoms presentation and then explored differences in suicide-related outcomes (i.e. suicide plans, suicide attempts) by the resulting classes.

**Results:**

A four-class model was determined to optimize the fit criteria. Class 3 (high depressive symptoms) had significantly greater rates of suicide-related outcomes, followed by class 1 (high depressed mood and moderate worthlessness), with classes 4 and 2 having significantly lower rates of suicide-related outcomes.

**Conclusions:**

The use of LCA provided valuable findings on the importance of leveraging both a multi-faceted assessment of depressive symptoms to identify cases where a high number of depressive symptoms are endorsed, and review of the specific symptoms endorsed. Worthlessness, in particular, may be of particular value to focus on within the context of suicide prevention.

Unipolar major depressive disorder (MDD) is a strikingly common and often disabling disorder, representing a leading cause of illness and contributor to suicide in the United States (Schiller & Norris, 2023). In the Diagnostic and Statistical Manual of Mental Disorders 5-TR (DSM-5-TR), MDD is characterized by at least five out of the nine following symptoms: depressed mood, anhedonia, significant weight loss or gain, insomnia or hypersomnia, psychomotor disturbance, fatigue, feeling worthless, decreased concentration, and thoughts of death or suicide (APA, 2022). At least one of the five symptoms must be depressed mood or anhedonia. Accordingly, MDD represents a remarkable heterogeneous disorder, with over 250 unique symptom combinations that meet DSM-5-TR diagnostic criteria (Buch & Liston, 2021; Zimmerman, Ellison, Young, Chelminski, & Dalrymple, 2015). Concerns regarding the heterogeneity of depression have long been raised, often supported by the fact that considerable variation exists in patient profiles within a single diagnosis, resulting in difficulty to identify optimal treatments (Goldberg, 2011). Notably, depressive symptoms also have considerable content variability with some symptoms focused on psychosomatic features (e.g. appetite changes, sleep changes), whereas other symptoms are cognitive (e.g. worthlessness), with others specific to mood (e.g. depressed mood), indicating the importance of examining depression on a symptom-level.

In efforts to better understand depression symptomatology, numerous studies have sought to develop symptom classes and profiles that can aid clinicians in identifying and treating particular profiles of patients presenting with MDD (e.g. Fried and Nesse, 2015). For instance, Wang et al. (2021) sought to establish associations between functional network connectivity and clinical symptom profiles, leveraging *K*-means clustering to develop distinct patient subtypes. Such efforts highlight the necessity of understanding how particular sets of symptoms may group together in informative ways. Similarly, other research has used depressive symptom profiles to understand treatment response (González-Roz, Secades-Villa, García-Fernández, Martínez-Loredo, & Alonso-Pérez, 2021) and to predict neurodegenerative diseases (Shdo et al., 2020).

Importantly, this existing body of research on depressive symptom profiles highlights that some depressive symptoms are more predictive of clinical outcomes than others. In a study of whether certain individual symptoms are more associated with psychosocial functioning during a course of psychotherapy, researchers found that changes in depressed mood, problems with sleep, fatigue, and anhedonia in particular were associated with improved functioning during treatment, with the strongest association for depressed mood (Malkki, Rosenström, Jokela, & Saarni, 2023). With this in mind, it is necessary to consider how specific depressive symptom profiles impact other clinical outcomes, such as suicide-related outcomes. While prior research has established various profiles, many of these symptom classes vary based on the outcome of interest and the population. Accordingly, it is important to determine whether existing profiles are replicated, such that existing profiles are generalizable across contexts, or if distinct profiles are more predictive of suicide-related outcomes. With hundreds of unique depression symptom profiles, it is essential to not rely exclusively on existing profiles developed for specific populations and outcomes, and instead consider new empirically derived profiles specific to a given outcome.

In addition to thoughts of death and suicide serving as a symptom of MDD (APA, 2022), suicide risk has long been linked to depression, with evidence highlighting the considerable, though non-total overlap between depression screening and suicide risk screening among adult samples, with 69% of patients who screened positive for suicide risk, also screening positive for depression (Mournet et al., 2021). Despite greater clinical overlap in depression and suicide risk, limited research to date has sought to investigate whether patients with particular groups of depressive symptoms are more likely to report suicide plans and attempts, compared to patients with other depressive symptom profiles. To address this gap, this secondary analysis leverages 7 years of data (2008–2014) from the National Survey on Drug Use and Health (NSDUH; Substance Abuse and Mental Health Services Administration [SAMHSA], 2016). In particular, we use latent class analysis (LCA) of depressive symptoms to explore differences in suicidal thoughts, plans, and attempts by class among adults endorsing depressive symptoms.

## Methods

### Study design and participants

This study utilized data from the 2008–2014 NSDUH, which was administered by SAMHSA's Center for Behavioral Health Statistics and Quality (SAMHSA, 2016). This survey is of a nationally representative sample of the US civilian, noninstitutionalized population and employs a multistage area probability sampling method. Given the use of secondary data, this study was deemed exempt from IRB review. Informed consent was obtained during original data collection. The NSDUH study is a cross-sectional study, collecting data on mental and behavioral health, including depressive symptoms. The present analyses contain only adults ages 18 years or older. Items were administered by module, with branching logic leading to only participants who endorse depressed mood and/or anhedonia completing the depressive items (described below). A total of 46 978 individuals were administered the depressed mood and anhedonia symptom items. Of these individuals, 43 023 endorsed at least one depression symptom. Individuals with missing data (2.4%; 1054/43 023) for the variables utilized in the current analyses were excluded, resulting in final sample of 41 969. Participant demographic characteristics are reported by wave in Table 1.

**Table 1.** Participant demographics

Variable	*N* (%)
Sample size	41 969
NSDUH collection year	
2008	5788 (13.79%)
2009	5941 (14.16%)
2010	5948 (14.17%)
2011	5959 (14.20%)
2012	6017 (14.21%)
2013	5945 (14.17%)
2014	6371 (15.18%)
Age	
18–25 years old	19 880 (47.37%)
26–35 years old	7004 (16.69%)
36–64 years old	12 886 (30.70%)
65 + years old	1199 (2.86%)
Sex	
Female	27 382 (65.24%)
Male	14 587 (34.76%)
Race/ethnicity	
Non-Hispanic White	29 374 (70.00%)
Non-Hispanic Black/African American	3759 (8.96%)
Non-Hispanic Native American/Alaskan Native	590 (1.41%)
Non-Hispanic Native Hawaiian/other Pacific Islander	168 (0.40%)
Non-Hispanic Asian	1165 (2.78%)
Non-Hispanic more than one race	1654 (3.94%)
Hispanic	5259 (12.53%)

### Measures

#### Demographics

Participants provided their date of birth, which is used to compute the age variable used in the NSDUH database. NSDUH categorizes adult ages into age ranges (e.g. ‘Respondent is between 50 and 64 years old’). For sex, participants were able to select ‘Female’ or ‘Male’. For race and ethnicity, participants were asked a single item containing a set of seven race/ethnicity categories. Table 1 provides all response options.

#### Depressive symptoms and suicide outcomes

All adult participants began the adult depression module with the item ‘Have you ever in your life had a period of time lasting several days or longer when most of the day you felt sad, empty or depressed?’ Using a series of branching logic, participants who endorsed ever experiencing depressed mood or anhedonia were asked a series of items to determine recent experience of each of the nine DSM-5-TR defined depressive symptoms (depressed mood, anhedonia, appetite/weight changes, sleep disturbances, psychomotor disturbance, fatigue, worthlessness, concentration issues, thoughts of death/self-harm). For some symptoms, participants were asked multiple items to assess different aspects of the symptoms. For all items participants were asked to think both about the period of time where symptoms were the most recent as well as the period where they were the worst. If a participant endorsed either, they were coded as endorsing the symptom. For instance, for worthlessness, participants were asked ‘Did you feel totally worthless nearly every day?’ The depressive symptom variables used in the present analyses are AD_MDEA1 through AD_MDEA8. Complete branching logic and all items are published elsewhere (Center for Behavioral Health Statistics and Quality, 2022). Individuals who endorsed suicidal thoughts earlier in the adult depression model were asked ‘Did you think about committing suicide?’. Participants who responded yes were asked: ‘Did you make a suicide plan?’ to assess suicide plans and ‘Did you make a suicide attempt?’ to assess suicide attempts. For those who responded ‘No’ to the suicidal thought item, a ‘No’ response was imputed both for suicide plans and for suicide attempts.

### Statistical analyses

We used LCA to identify subgroups within our sample based on depressive symptoms using the *tidySEM* package in R (Van Lissa, Garnier-Villarreal, & Anadria, 2023). LCA is a statistical approach that is used to identify qualitatively distinct groups within a given sample that share certain characteristics, such as the presence of symptoms. The depressive symptom items, except for the ninth item focused on thoughts of death or urges to self-harm, were set as the indicator variables for the LCA. The ninth item was not used as an indicator given its overlap with the suicide-related outcomes of interests to avoid this item conflating associations. Several iterations of the LCA were run with increasing numbers of classes to identify which model was optimal. To determine which model was optimal, we considered several indicators of fit, prioritizing low Bayesian information criterion (BIC) among models where all class population shares were optimal at >5% (Van Lissa et al., 2023; Zhang, Abarda, Contractor, Wang, & Dayton, 2018). We compared models with *k* classes to a model with *k–1* classes to determine if our model improved as a result of adding an additional class. After identifying the best-fitting latent class model, we used the Bolck, Croon, and Hagenaars (2004; BCH) method to examine whether particular classes were more likely to report suicide plans and attempts, compared to other classes. This method reduces bias by accounting for classification error and class membership while examining distal outcomes (Vermunt, 2017).

## Results

Table 2 provides descriptive statistics of the fit criteria that were used to determine the optimal number of latent classes. We analyzed models with up to five classes and found that at five classes, population shares for select classes went below 5%. Accordingly, we opted to select the model with the lowest BIC that had no classes with population shares less than 5%, given the recommendation for class shares to be no smaller than 5% of the sample (Weller, Bowen, & Faubert, 2020). Based on this information, the four-class model was determined to optimize the fit criteria. Although there is no agreed upon standard cutoff for entropy, we do note that the entropy values across all models were at the limits of what is generally accepted (Weller et al., 2020). In the four-class model, 57.5% of the sample was included in the third class, followed by 27.3% in the fourth class, 8.2% in the first class, and 6.9% in the second class. The rate of endorsement of each class for each depressive symptom, suicidal ideation, suicide plans, and suicide attempts is provided in Table 3.

**Table 2.** Class descriptive statistics

Classes	Log likelihood	Parameters	AIC	BIC	Entropy	Class share minimum %
1	−144 786.6	8	289 589.2	289 658.3	1.000	n/a
2	−135 393.4	17	270 820.8	270 967.7	0.626	0.31
3	−134 470.1	26	268 992.3	269 217.0	0.587	0.08
4	−134 240.0	35	268 549.9	268 852.5	0.594	0.07
5	−133 572.1	44	267 232.2	267 612.6	0.714	0.03

**Table 3.** Rates of endorsement of depression symptoms and suicide-related outcomes by class

Variable	Class 1	Class 2	Class 3	Class 4
Depressed mood	1.00	0.00	0.98	1.00
Anhedonia	0.61	1.00	0.99	0.76
Appetite	0.58	0.77	0.95	0.79
Sleep	0.46	0.85	0.98	0.97
Activity	0.13	0.31	0.73	0.19
Fatigue	0.24	0.83	0.98	1.00
Worthless	0.24	0.08	0.80	0.13
Concentrate	0.41	0.60	0.98	0.59
Suicidal ideation	0.26	0.15	0.48	0.25
Suicide plan	0.07	0.03	0.17	0.07
Suicide attempt	0.05	0.02	0.14	0.04

**Figure 1.**
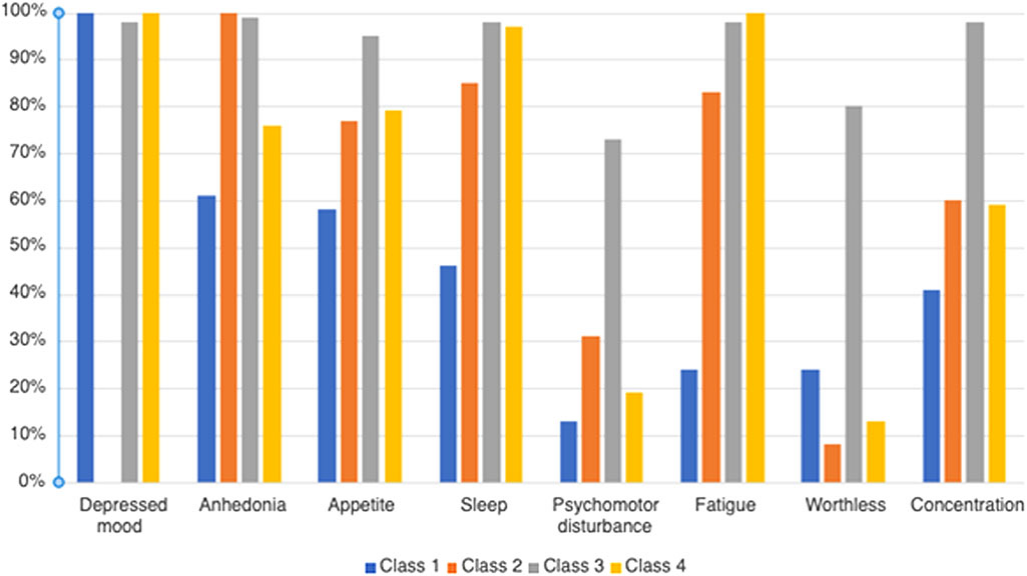
Bar chart of percentage of endorsement of depressive symptoms by class.

Qualitatively, class 1 (8.2% of sample) had high endorsement of depressed mood (100%), with low endorsement of other depressive symptoms. Relative to classes 2 and 4, class 1 also had moderately high worthlessness. Class 2 (6.9% of sample) was characterized by high endorsement of anhedonia (100%) and moderate endorsement of appetite-related symptoms, sleep difficulties, fatigue, and trouble concentrating. Class 3 (57.5% of sample) had moderate endorsement of worthlessness and psychomotor disturbance, with high endorsement of all other examined symptoms. Class 4 (27.3% of sample) had high endorsement of depressed mood, sleep difficulties, and fatigue, as well as moderate endorsement of anhedonia, appetite-related symptoms, and trouble concentrating. Figure 1 provides a bar chart of endorsement of depressive symptoms by class.

LCA via the BCH method were performed to examine differences in suicide-related outcome endorsement by latent class membership (Table 3). There were significant differences among classes for suicidal ideation (log likelihood difference [LL-dif] = 5463.846, df = 3, *p* < 0.001), suicide plans (LL-dif = 3492.824, df = 3, *p* < 0.001), and suicide attempts (LL-dif = 2735.97, df = 3, *p* < 0.001). Pairwise analyses revealed that class 3 had significantly higher rates of suicidal ideation, plans, and attempts, followed by classes 1, 4, and then 2, with significant differences between each class, with the exception of no difference between suicide attempt rates between classes 2 and 4 (see Table 4 for pairwise comparison tests by class).

**Table 4.** Pairwise comparison tests by class for suicide-related outcomes

Outcome	Class comparison	Log likelihood difference	*p*-value
Suicidal ideation	Class 1 > 2	222.702	<0.001
	Class 1 < 3	1127.400	<0.001
	Class 1 > 4	142.037	<0.001
	Class 2 < 3	1879.758	<0.001
	Class 2 > 4	51.715	<0.001
	Class 3 > 4	4029.745	<0.001
Suicide plans	Class 1 > 2	155.433	<0.001
	Class 1 < 3	622.383	<0.001
	Class 1 > 4	165.847	<0.001
	Class 2 < 3	1005.816	<0.001
	Class 2 > 4	18.175	<0.001
	Class 3 > 4	2612.878	<0.001
Suicide attempts	Class 1 > 2	81.502	<0.001
	Class 1 < 3	466.678	<0.001
	Class 1 > 4	175.971	<0.001
	Class 2 < 3	668.306	<0.001
	Class 2 = 4	0.021	0.885
	Class 3 > 4	2149.234	<0.001

Note: df = 1 for all comparison tests.

## Discussion

The present findings revealed a unique class structure that was found to have significant differences with regards to endorsement of suicide plans and attempts among this sample of adults endorsing depressive symptoms. Such information can be leveraged by mental health providers to improve identification of individuals at risk for suicide, using depression symptomatology. Of note, one class was characterized by high endorsement of depressive symptoms (class 3). With emergence of a class that had consistently high symptom endorsement, regardless of the symptom, results highlight the importance of considering a composite depression symptom score as a way to measure the number of endorsed symptoms. Importantly, class 3, which consisted of participants endorsing high levels of all symptoms, was most likely to also endorse suicide-related outcomes. These analyses highlight that in this adult sample, endorsement of more depressive symptoms is associated with higher risk of suicide-related outcomes. This is consistent with existing research highlighting that greater depression symptom severity and variability across symptoms was predictive of suicide attempts (Melhem et al., 2019). Previous studies that have leveraged mixture modeling of depressive symptoms have similarly found that some detected symptom groups are characterized by symptom severity more so than specific symptoms (González-Roz et al., 2021). This has important implications for clinical practice. Namely, when screening for depression, clinicians who find clients endorsing many symptoms should consider also screening for risk of suicide.

Beyond the value of a composite score of total depressive symptoms, results highlight the importance of examining the individual items endorsed by a person. Unique symptom classes emerged in classes 1, 2, and 4. Of note, a major difference in the characterization of classes 1, 2, and 4 is that there is moderate endorsement of worthlessness in class 1 compared to classes 2 and 4 which had particularly low worthlessness. Class 1 had the second highest rate of suicidal ideation, plans, and attempts. This finding may point to the symptom of worthlessness as being a key factor in the class 1 group of symptoms. This is in line with prior research using the National Epidemiologic Survey on Alcohol and Related Conditions database which found that among participants experiencing a major depressive episode, worthlessness was the only depressive symptom that was a significant indicator of elevated risk of suicide attempts after remittance of the depressive episode (Wakefield & Schmitz, 2016). Taken together, the results of the current study and prior research underscore a need for mental health providers to pay close attention to worthless among depressed adults as a means to preventing suicide.

These symptom classes are unique and in contrast to existing research on depression symptom classes and profiles. For example, a previous study using self-report and MRI data found two depression symptom subtypes, one characterized by anhedonia and one characterized by insomnia (Wang et al., 2021). Another study of patients with neurodegenerative diseases detected profiles consisting of hopeless, dysphoric, and withdrawn pattern but varying levels of severity of symptoms (Shdo et al., 2020). Results from the present analyses may differ given the use of a sample of people with depressed mood and anhedonia. Moreover, the findings of the current study highlight the importance of examining symptom groups in relation to specific clinical outcomes and populations. This information has the ability to provide valuable clinical information to mental health providers on features to pay attention to when seeking to understand suicide risk among clients.

Although this study contains many strengths, including a large, nationally representative sample of adults, there are several important limitations to note. Namely, due to the use of branching logic in the NSDUH survey, participants who did not endorse experiencing any level of depressed mood or anhedonia in their lifetime were not administered the subsequent depression module items. Accordingly, due to this skip logic, this sample represents a sample of adults with a history of depressive symptoms, limiting generalizability to adults without a history of depressive symptoms. Nonetheless, these analyses represent an important step in continuing to advance the understanding of the link between depression and suicide. The present analyses should be replicated among a sample of individuals with greater variability in depressive symptom history to better understand suicide outcomes among non-depressed individuals. An additional limitation to note was the exclusion of the ninth depressive symptom, thoughts of death or self-harm. Given overlap in this suicidal ideation focused depressive symptom and the suicide-related outcome of suicidal ideation, the present analyses sought to focus on non-suicidal ideation predictors and outcomes. Finally, it is important to note that this dataset utilizes the phrase ‘committing suicide’ to query for suicidal ideation. While we retained this language for the purposes of data integrity, it is essential to consider that the American Foundation for Suicide Prevention (AFSP) safeguard recommendations advise against the use of ‘committing’ in relation to suicide (AFSP, 2024). This guidance should be utilized in the development of future research studies on this topic.

The use of LCA provided valuable findings on the importance of leveraging both a composite score of depressive symptoms to identify cases where a high number of depressive symptoms are endorsed, and review of the specific symptoms endorsed. The highlighted results have important clinical implications. Worthlessness, in particular, may be of particular value to study further in the context of suicide prevention. Moreover, in both research and clinical settings, considering total symptoms endorsed and the specific items endorsed for each client has the potential to aid in the identification of risk of suicide. Future research should continue to identify how classes of depressive symptoms may be predictive of clinical outcomes.
